# Admission neutrophil-to-lymphocyte ratio to predict mortality in burn patients: a meta-analysis

**DOI:** 10.1186/s40635-024-00668-x

**Published:** 2024-10-01

**Authors:** Mohamed K. A. Awad, Ahmed Elsahhar, Mahmoud Alwakeel, Radwa Awad, Nada Gomaa, Amr Muhammad Abdo Salem, Mahmood Ramadan, Ghada Elsahhar, Reyad Essam Reyad Abdelbaky, Francois Fadell

**Affiliations:** 1 Department of Anesthesia and Critical Care, Faculty of Medicine, Ain Shams University, Cairo, Egypt; 2 Department of Pulmonary and Critical Care Medicine, Respiratory Institute, Cleveland Clinic, 9500 Euclid Ave, Mail Code A90, Cleveland, OH 44195 USA; 3 Department of Critical Care, Cairo University, Cairo, Egypt; 4 Department of Radiology, Faculty of Medicine, Ain Shams University, Cairo, Egypt; 5 Egyptian Ministry of Health, Cairo, Egypt; 6 General Surgery Department, Hereford County Hospital (Wye Valley NHS Trust), Hereford, UK; 7 Veterans Affairs Western New York Health Care System, 3495 Bailey Avenue, Buffalo, NY 14215 USA; 8 Jacobs School of Medicine, University at Buffalo, Buffalo, NY USA; 9 Birmingham Women’s and Children’s NHS Foundation Trust, Birmingham, UK

**Keywords:** Burn, NLR, Neutrophil-to-lymphocyte ratio, Mortality

## Abstract

**Background:**

The neutrophil-to-lymphocyte ratio (NLR) proves to be a convenient and cost-effective marker with studies showing that a high NLR can serve as a mortality indicator in burn cases. We conducted a meta-analysis aiming to explore whether on-admission NLR values could serve as predictors of mortality in burn patients.

**Methods:**

PubMed, Web of Science, Scopus and Embase were searched from inception until January 2024. We included all studies investigating burn patients that contain information on the NLR value at the time of hospital admission and mortality outcomes. The studies were critically appraised using the NIH Quality Assessment Tool.

**Results:**

Nine studies fulfilled our criteria with a total population of 1837 participants, including 1526 survivor Burn patients and 311 non-survivor Burn patients. The overall mean difference measured by random model showed a significant increase in NLR by 5.06 (95% CI 3.42, 6.68) *p* ≤ 0.001 for the non-survivor group over the survivors group with heterogeneity *I*^2^ = 67.33%, *p* ≤ 0.001. A meta-regression was done to investigate the potential source of heterogeneity among studies. The results showed that age (*p* = 0.394), gender (*p* = 0.164), and sample size (*p* = 0.099) did not contribute to the source of heterogeneity, however, the burn surface area contributed significantly (*p* = 0.002). A leave-one-out meta-analysis was done, showing that omitting Le Qui et al., leads to significantly decrease the heterogeneity to be *I*^2^ = 2.73%. Meta-regression repeated to assess the burn surface area again to be found noncontributing (*p* = 0.404).

**Conclusions:**

Our findings support that elevated NLR values can serve as a mortality indicator in burn cases. This will have a great clinical impact by aiding in stratifying the burn patients on admission.

## Background

Burn injuries rank as the fourth most prevalent form of trauma globally [1]. The World Health Organization reports that ~ 180,000 deaths occur annually worldwide due to burns, with a predominant occurrence in low- and middle-income countries [2].

A systemic inflammatory response is activated by thermal injuries, and it is thought to significantly contribute to the pathophysiology of the primary disturbances observed in individuals with burn injuries [3]. Traditionally, a range of clinical and laboratory indicators has been employed to assess the prognosis of burn patients. These include burn injury severity scores, RYAN score, R-BAUX score, as well as inflammatory markers such as erythrocyte sedimentation rate (ESR), C-reactive protein (CRP), and procalcitonin (PCT) [4–6].

As the quest for new inflammatory and prognostic markers persists, it becomes imperative to identify more readily accessible parameters, particularly given that 90% of deaths related to burns occur in low- and middle-income countries [1]. Neutrophils, which accumulate in organs due to the systemic inflammatory response following burn injuries, serve as the primary generators of free oxygen radicals. Additionally, there is an inhibition of the cellular immune response, leading to a decrease in delayed-type hypersensitivity reactions and lymphocyte count in peripheral blood [7].

The neutrophil-to-lymphocyte ratio (NLR) is recognized as a marker of systemic inflammation and has been associated with disease severity and survival across various conditions such as cancer, heart failure, sepsis, and acute respiratory distress syndrome [8–13]. The NLR is determined by dividing the absolute neutrophil count by the absolute lymphocyte count [14].

Significant burn injuries result in various changes in complete blood count (CBC) following admission [15, 16]. Likely influenced by several factors, such as hemodilution due to fluid resuscitation and bone marrow depression [17], the use of a ratio proves beneficial in illustrating the relative alterations in CBC parameters.

Numerous studies have reported the prediction of burn patient mortality based on the admission NLR [17–23]. Conducting a systematic review and meta-analysis, we aimed to explore whether on-admission NLR values could serve as predictors of mortality in burn patients.

## Methods

### Search strategy

This analysis was conducted in line with the Preferred Reporting Items for Systematic Reviews and Meta-Analyses guidelines [24].

**Fig. 1:**
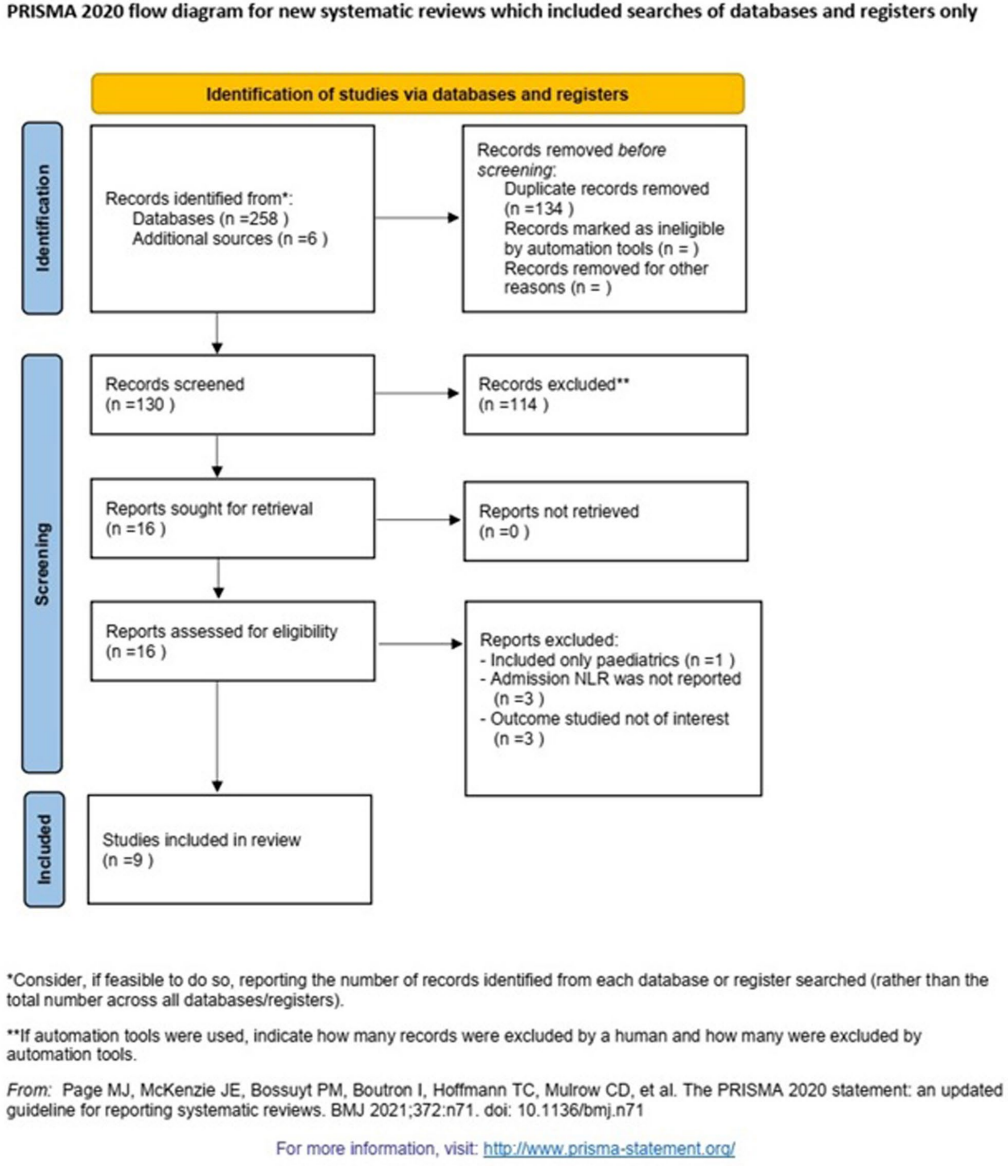
PRISMA flowchart

PubMed, Web of Science, Scopus and Embase were systematically searched to identify suitable articles published until January 2024. The search terms included: (neutrophil to lymphocyte) OR (NLR) OR (neutrophil-to-lymphocyte) AND (Burn) AND (mortality). We searched for articles in all languages, which were translated when necessary. Articles were also identified using the “related articles” function in PubMed and by manually searching the references within identified articles. The full search strategy is described in Fig. 1.

### Eligibility criteria

We included all research papers investigating burn patients that contain information on the NLR value at the time of hospital admission and mortality outcomes. The following articles were excluded from this review: non-research letters, correspondences, case reports, review articles, animal studies and studies that included only pediatrics.

### Study selection

All articles obtained through systematic searches of electronic databases were transferred to EndNote 20 bibliographic and reference manager. Rayyan software was employed to eliminate duplicates. The titles and abstracts underwent independent screening by two reviewers, and potentially relevant articles were further assessed for eligibility against the predefined criteria. Any discrepancies were resolved through discussion until a mutual consensus was achieved.

### Data extraction

Three reviewers separately gathered data from the included papers. Before the data extraction process, a customized, standardized form for data extraction was created. The extracted data encompassed various elements, including the first author, year of the study, publication type, study location, study design, baseline population characteristics, exposures, and outcomes.

In this context, exposure was defined as the NLR value upon admission to the hospital, presented in terms of continuous NLR values. The primary outcome of interest was mortality. The data initially presented as median and IQR were transformed into mean and SD using established tools from previous publications [25, 26].

### Quality assessment

The studies were critically appraised using the NIH Quality Assessment Tool for Observational Cohort and Cross-Sectional Studies and the NIH quality assessment tool for observational case–control studies by three independent reviewers, and when there was a discrepancy in the assessment score, discussions were done to reach an agreement [27].

### Statistical analysis

The meta-analysis was performed using the Stata 18 software. Continuous variables were expressed as MD (mean difference), with each effect size expressed as a 95% confidence interval (CI). The value of *I*^2^ < 50% indicated the lack of heterogeneity across the studies, when statistical heterogeneity was indicated; Meta-regression was done to detect source. Sensitivity analysis was done using a leave-one-out meta-analysis to show how each individual study affects the overall estimate by removing one study alternately from the meta-analysis. Publication bias was assessed qualitatively using the funnel plot and quantitatively using Egger's linear regression test to evaluate the presence of small-study effects. A meta-regression was performed for the following potential confounders: age, gender, burn area and sample size. A statistically significant difference was considered if a two-tailed *p* < 0.05.

## Results

### Search results

Our search strategy resulted in a total number of 264 studies. After the title and abstract screening and removing the duplicates, 134 articles were eliminated, and 16 full-text articles were evaluated for eligibility. Following the full-text screening, 9 papers [17–23, 28, 29] met our criteria and were included in our systematic review and meta-analysis (Fig. 1).

### NLR and mortality

We included 9 studies (6 cross sectional, 2 retrospective cohort and 1 case control) with a total population of 1837 participants, including 1526 survivor burn patients and 311 non-survivor burn patients. The mean age of participants was 38.15 year. Of which, 1284 were males, and 557 were females. The main characteristics of the included studies are summarized in Table 1.

**Table 1:** Study characteristics

Author	Country	Sample size *N*	Type of study	Age Mean ± SD/median (IQR)	Male *N* (%)	Degree of burn	Type of burn	Burn area Mean ± SD/median (IQR)	NLR Mean ± SD/median (IQR)
Ciftci 2019	Turkey	Total 366 Survive 314 Died 52	Cross sectional	28.66. ± 21.12 26.31. ± 19.95 42.85. ± 22.64	268 (73.2%) 229 (72.9%) 39 (52%)	2nd and 3rd degree burn	Not reported	23.65 ± 18.80 18.95 ± 12.74 52 ± 24.02	5.54 ± 5.6 5 10.94 ± 7.63
Angulo 2020	Uruguay	Total 88 Survive 75 Died 13	Cohort	47 (28–60) 43 (26–59) 52 (40–75)	62 (70.4%) 52 (69.3%) 10 (76.9%)	3rd degree burn, Inhalation injury	Thermal and inhalation injuries	14 (7–23) 11 (6–19) 44 (30–66)	8.7 [4.8–12.4] 15.0 [9.7–25.7]
Bhuyan 2020	India	Total 242 Survive 194 Died 48	Cross sectional	34.38 (Mean) 46.82 (Mean)	152 (62.8%) 127 (65.4%) 25 (52%)	Inhalation injury, 2nd and 3rd degree burn	Not reported		7.23 ± 3.25 14.44 ± 6.95
Temiz 2020	Turkey	Total 133 Survive 109 Died 24	Cross sectional	15.17 ± 18.23 33.04 ± 26.64	69 (51.8%) 61 (55.9%) 8 (33.3%)	1st, 2nd and,deep burn	Scald, flame, and electric burn	22.92 ± 9.11 52.04 ± 23.52	6.34 ± 12.13 12.96 ± 9.70
Steinvall 2021	Sweden	Total 222 Survive 185 Died 37	Cohort	55 (38–69) 52 (37–66) 69 (63–74)	148 (66.7%) 127 (68.6%) 21 (56.8%)	Superficial, deep, full thickness	Not reported	24.5 (13–37.2) 21.5 (12.5–32.5) 43 (32–63)	9.72 (5.38–16.16) 8.75 (5.19–14.67) 12.65 (7.46–18.64)
Le Qiu 2021	China	Total 577 Survive 522 Died 55	Cross sectional	43.58 ± 15.11 52.55 ± 17.98	384 (73.6%) 41 (74.5%)	TBSA ≥ 30%, full-thickness burn ≥ 10%, inhalation	Flame, scalding, electric, contact, and inhalation	49.59 ± 17.94 67.49 ± 25.07	14.45 ± 9.46 15.30 ± 7.99
Setwani 2022	Indonesia	Total 60 Survive 30 Died 30	Case control	38.5(30.5–52.5) 37.5 (31–48) 39.5(30–63)	18 (30.0%) 10 (33.3%) 8 (26.7%)		Flame, scald and electric	34.8 (26–46) 29.3 (23.5–34.5) 46.0 (36–65)	15.6 (10.1–21.7) 13.2 (9.9–16.9) 21.4 (12.4–28.6)
Lesmanawatia 2023	Indonesia	Total 126 Survive 84 Died 42	Cross sectional	26.9 ± 18.3 43.17 ± 18.19	88 (69.8%) 60 (71.4%) 28 (66.67%)		Flame, scald, chemical, hot stream	22.78 ± 12.89 53.11 ± 23.11	7.92 ± 5.98 12.05 ± 9.74
Guzmán 2023	Germany	85 Survive 67 Died 18	Cross sectional	40 ± 17.4 37.27 ± 16.3 50.17 ± 17.98	69 (81.2%) 55 (79.7%) 14 (20.3%)		Electric, flame, scald and chemical		10.64 ± 7.7 9.73 ± 7.7 13.4 ± 6.9

**Fig. 2:**
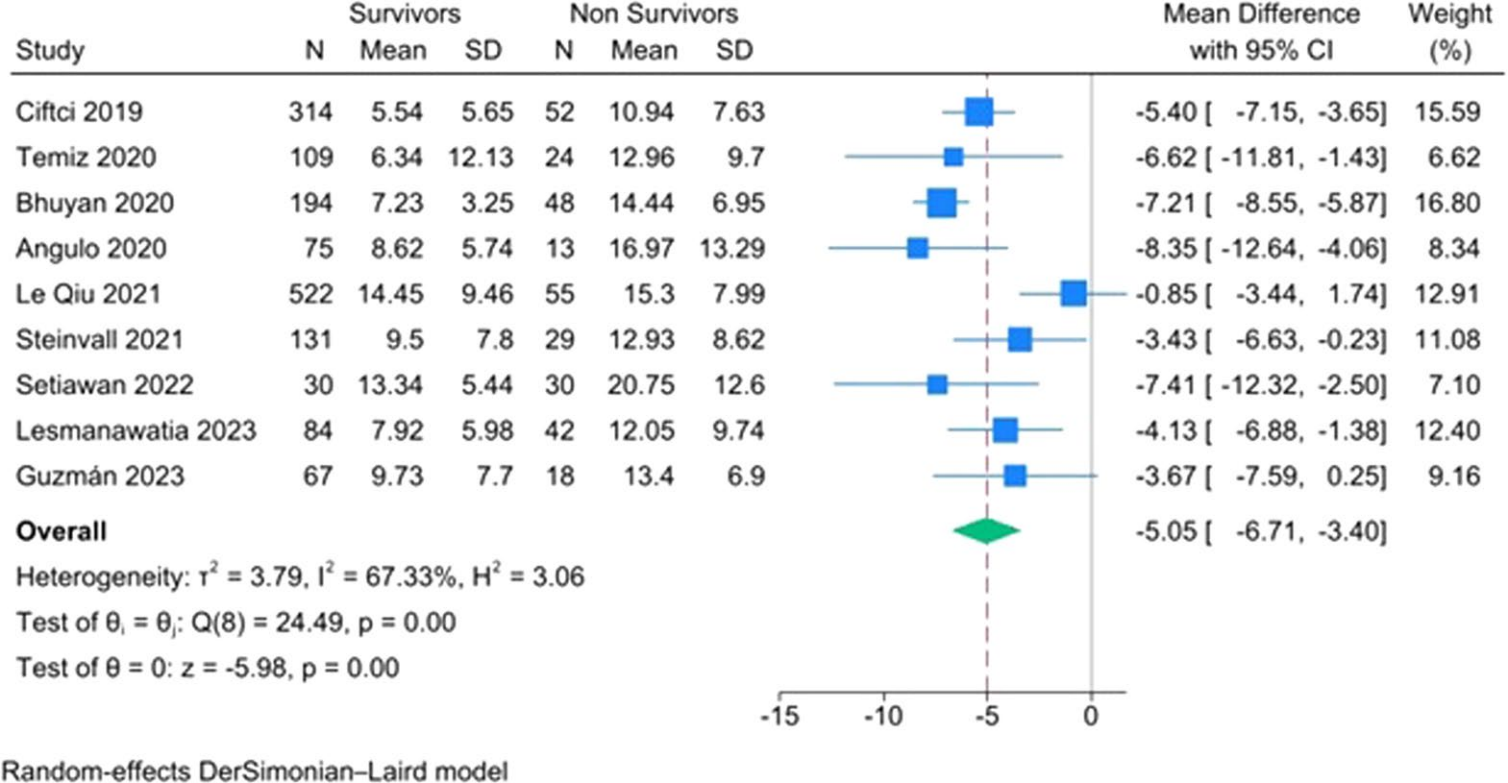
Forest plot of NLR

The overall mean difference showed a significant increase in NLR by 5.06(95% CI 3.42, 6.68) *p* ≤ 0.001 for the non-survivors group over the Survivors group with heterogeneity *I*^2^ = 67.33%, *p* ≤ 0.001 (Fig. 2).

A meta-regression was done to investigate the potential source of heterogeneity among studies. The results showed that age (*p* = 0.394), gender (*p* = 0.164), and sample size (*p* = 0.099) did not contribute to the source of heterogeneity; however the burn surface area contributed significantly (*p* = 0.002).

**Fig. 3:**
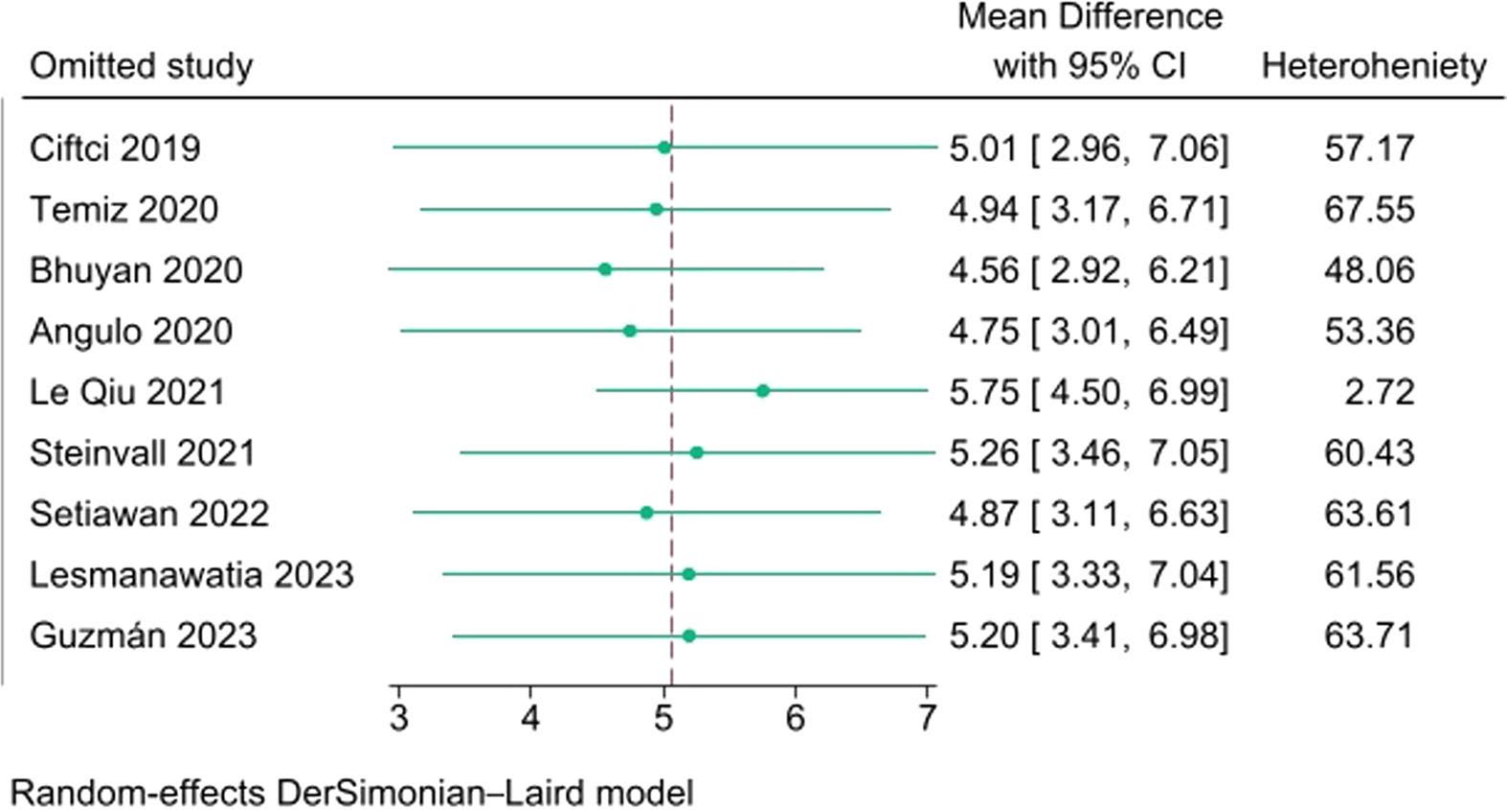
Leave-one-out meta-analysis

A leave-one-out meta-analysis was done (Fig. 3), showing that omitting Le Qui et al. [29] leads to significantly decrease the heterogeneity to be *I*^2^ = 2.7%. Meta-regression repeated to assess the burn surface area again to be found noncontributing (*p* = 0.404).

### Publication bias

**Fig. 4:**
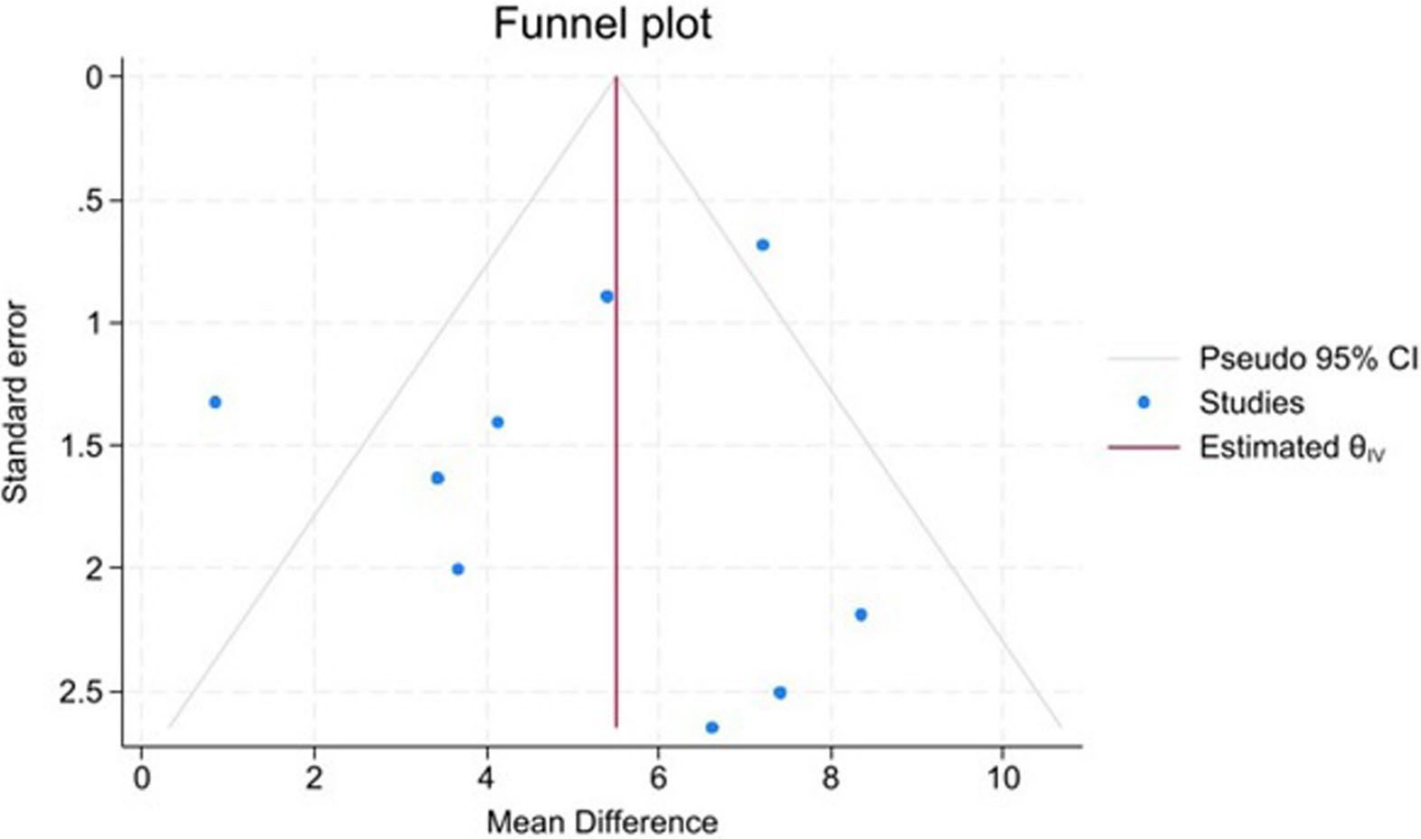
Funnel plot

Visual inspection of the funnel plot of the first meta-analysis did not show asymmetry, as shown in Fig. 4, Egger’s test showed that publication bias was statistically insignificant (*p* = 0.7371).

### Quality assessment

**Fig. 5:**
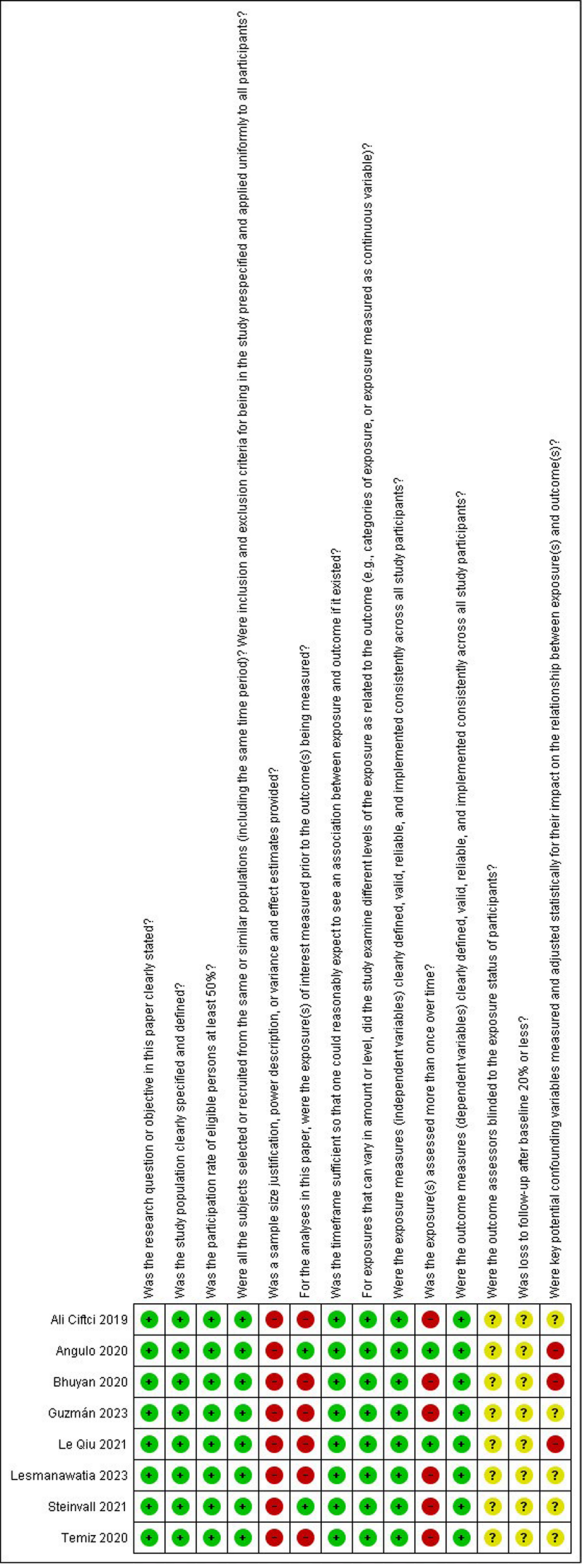
NIH quality assessment tool for observational cohort and cross-sectional studies. Total scores: yes = 1/no = 0.5/NR and NA and CD = 0. Quality rating: good (11–14 points), fair (7.5–10.5 points), or poor (0–7 points)

**Fig. 6:**
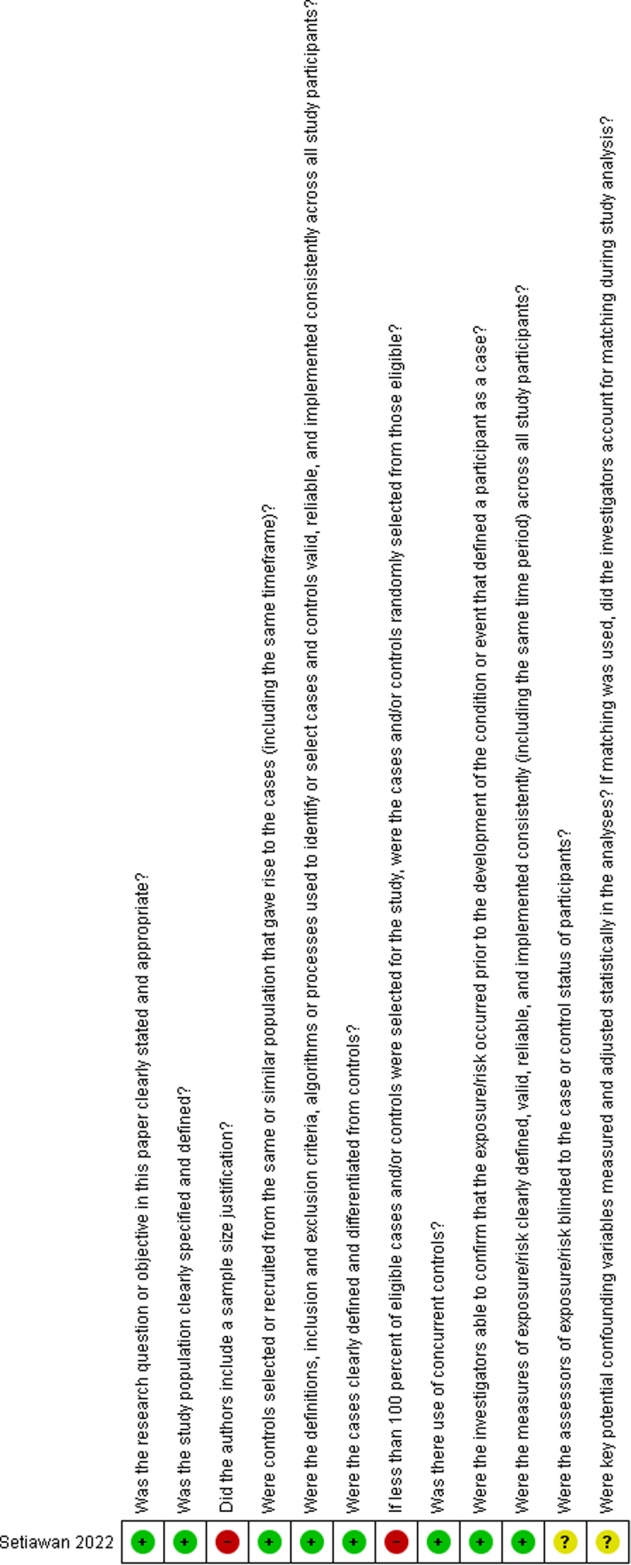
NIH quality assessment tool for observational case control studies. Total scores: yes = 1/no = 0.5/NR and NA and CD = 0. Quality rating: good (9.5–12 points), fair (6.5–9 points), or poor (6–0 points)

For the cohort and cross-sectional studies reviewed by the NIH tool, one was good quality, and seven were fair quality and for observational case–control study reviewed by the NIH tool, the study was fair (Figs. 5, 6).

## Discussion

Burn injuries trigger the activation of complex mediator systems, resulting in pathophysiological changes within the body [30]. Various biochemical values have been utilized as prognostic factors for sepsis and mortality in burn patients [31]. The neutrophil-to-lymphocyte ratio (NLR) proves to be a convenient and cost-effective marker, readily available without the need for additional laboratory testing. Previous research has demonstrated that a high NLR is associated with increased morbidity and prolonged hospital stays [32], indicating its potential as an indicator of the inflammatory process. Accumulating evidence from several studies suggests that an elevated NLR can serve as a mortality indicator in burn cases.

While several studies, including ours, have evaluated the use of NLR as a prognostic factor in burn patients, a meta-analysis specifically focusing on the admission neutrophil-to-lymphocyte ratio in predicting mortality in burn patients has not been conducted to our knowledge. All studies included in our meta-analysis were retrospective.

Our analysis, incorporating nine studies and a total of 1837 patients (1526 survivors and 311 non-survivors), revealed that the mean admission NLR was significantly higher in non-survivors compared to survivors. The overall mean difference indicated a significant increase in NLR by 5.06 (95% CI 3.42, 6.68), with a *p*-value of < 0.001 for the non-survivors group over the survivors group. However, there was heterogeneity observed (*I*^2^ = 67.33%, *p* ≤ 0.001). A leave-one-out meta-analysis demonstrated that omitting the study by Le Qui et al. [29] led to a significant decrease in heterogeneity to *I*^2^ = 2.73%.

The observed heterogeneity introduced by Le Qui et al. may be attributed to their specific inclusion criteria. Notably, they excluded patients who died within the first 7 days of admission, potentially overlooking cases with high neutrophil-to-lymphocyte ratio (NLR) in the early phase, which might have otherwise contributed to the mortality group. Additionally, their study incorporated individuals with the highest percentage of total body surface area (%TBSA) of ≥ 30%, further influencing the composition of their participant cohort.

Neutrophils, accumulating in organs due to the systemic inflammatory response triggered by burn injuries, serve as the primary source of free oxygen radicals, inducing tissue damage. There is also a suppression in cellular immune response results in reduction of lymphocyte count in the peripheral blood [7]. This process aligns with our findings that demonstrated higher NLR values in the non-survivor group. The results suggest that on-admission NLR can function as a prognostic factor for burn patients, as it reflects the systemic inflammatory response and correlates with adverse outcomes.

In a meta-analysis by Huang et al., it was suggested that neutrophil-to-lymphocyte ratio (NLR) could serve as a prognostic biomarker in sepsis patients, indicating poor outcomes for those with elevated NLR levels. It is worth noting that this conclusion, although applicable to sepsis, was not specific to burn cases. Additionally, conditions like cachexia might not trigger an increase in neutrophils, leading to a potential false-negative interpretation of neutrophil values in predicting sepsis. Simultaneously, the inflammatory process could cause a decrease in lymphocyte levels. The NLR, considering both parameters, is considered more reliable than relying solely on neutrophil or lymphocyte levels alone [33].

Previous studies reported normal mean value of NLR across all ages as 1.65, with men having a mean of 1.63 and women 1.66 [14, 34].

The Baux score, a prediction model for mortality after acute burn injury, incorporates age and burn size (TBSA%). Two studies analyzing Baux score correlated with NLR values found that patients with higher NLR values were in the mortality group and exhibited persistently higher R-BAUX scores [20, 28].

Despite our study's contributions, it has several limitations. Firstly, all the studies included are retrospective, introducing potential biases. Secondly, some crucial individual information was not provided, limiting the ability to perform a more accurate analysis stratified by different risk factors. Comorbidities were not reported in any of the studies, as most excluded patients with preexisting chronic diseases. None of the studies documented wound culture during admission to exclude wound infection. Time until admission was reported in only one study [20]. Admission severity scores (SOFA and ABSI) were mentioned only once [17, 20], while the BAUX score was reported in only two studies [20, 28]. Additionally, there was insufficient data on treatment modalities. The type and degree of burns were not detailed in most studies, an important factor, such as the surface area of burns, was mentioned in only 7 out of our 9 studies as shown in Table 1. Three studies only reported the sensitivity and specificity of the NLR as a marker, but was not sufficient to perform a reliable diagnostic test accuracy [17, 21, 29].

Thirdly, the inclusion of four out of the eight studies conducted in Asian countries raises the potential for immune variability among diverse ethnic populations [35]. Future studies with larger, more homogeneous populations are essential for advanced assessment of the true role of NLR in predicting outcomes for burn patients.

## Conclusions

NLR was found to be a feasible marker for predicting outcomes for burn patients. We think it should be studied in combination with other clinical parameters to be more accurate and precise.

## Data Availability

The presented data in the manuscript are available from the authors with a reasonable request.

## Abbreviations

NLR: Neutrophil-to-lymphocyte ratio
NIH: National Institutes of Health
